# The impact of fluorinated graphene versus nanohydroxyapatite crystals on artificial white spot lesion microhardness and color change

**DOI:** 10.1186/s12903-025-06410-7

**Published:** 2025-07-02

**Authors:** Sarah Diaa Shaheen, Mona Rizk Aboelwafa

**Affiliations:** 1 Lecturer of Conservative Department, College of Oral and Dental Surgery, Misr University for Science and Technology (MUST), Giza, Egypt; 2 Department of Operative Dentistry, Faculty of Dentistry, Sinai University - Kantara branch, Ismailia, Egypt

**Keywords:** Fluorinated graphene oxide nanosheets (FG), Nano-Hydroxyapatite (nHA), Cross-Sectional microhardness (CSMH), White spot lesions

## Abstract

**Objective:**

To compare the impact of the fluorinated graphene oxide nanosheets (FG) and nanohydroxyapatite (n-HAp) with and without microabrasion pretreatment on the cross sectional microhardness (CSMH) and color change (ΔE_00_) of artificially established white spot lesions.

**Materials and methods:**

Demineralized eighty bovine enamel samples were splited into two groups (*n* = 40) based on the remineralizing material (n-HAp or FG), subsequently divided into two subgroups (*n* = 20) dependent on microabrasion pretreatment. The samples are remineralized for thirty days. ΔE_00_, elemental analysis employing energy-dispersive X-ray spectroscopy (EDX) along with surface features with scanning electron microscopy (SEM) was assessed. CSMH was measured at 50, 100, and 150 μm enamel depths. Statistical analysis was performed using ANOVA and post-hoc testing (*p* < 0.05).

**Results:**

FG had the highest CSMH at 100 and 150 μm depths while microabrasion with remineralizing has a highest CSMH at 50 μm. EDX revealed that FG-treated specimens had the highest Ca/P ratio (*p* < 0.001). FG had the highest ΔE_00_, whereas microabrasion with n-HAp showed the least ΔE_00_.

**Conclusions:**

FG provides greater aesthetic enhancement and remineralization than n-HAp. microabrasion did not significantly boost the remineralization.

**Clinical significance:**

Fluorinated graphene oxide nanosheets are a promising remineralization approach for treating white spot lesions offering improved mineral recovery and aesthetic outcomes.

## Introduction

White spot lesion (WSL) is a descriptive diagnostic terminology that denotes the earliest phase of demineralization in the enamel’s outer and deep layers, arising from plaque deposition in stagnant areas in persons with improper oral hygiene [1]. In recent years, the overall incidence of these lesions has increased, with estimates ranging from 10 to 49% [2]. If overlooked, these lesions may develop into a constant decline in minerals and disintegration until loss of surface continuity [3].

First-stage enamel lesions comprise a partially unaffected surface band that covers the extremely permeable and demineralized portion of the defect, termed the body of the lesion. Hypomineralized enamel has a more exceedingly porous and disordered structure than typical polycrystalline enamel, with a mineral content that decreased by 28%. The demineralization cycle produces mineral depletion from the enamel because of the hydroxyapatite disintegration in an acidic medium, which triggers enamel porosity. Therefore, the hypomineralized enamel’s hardness is considerably lower than that of sound enamel [4]. Clinically, the WSL seems opaque white, owing to the visual effects arising from mineral lack and the variant water and air refractive indices that occupy the voids produced inside the enamel [5, 6]. Moreover, there is a rise in the enamel pores and surface imperfections, resulting in brightness loss and diffused light reflection [7, 8].

Multiple interventions have been suggested for halting WSLs and concealing their whitish appearance. Furthermore, long term establishment of this aesthetic appearance might be demanding. A crucial aspect of minimal intervetion dentistry is the remineralization of WSLs rather than their restoration [9]. By encouraging the remineralization of the subsurface enamel lesions, remineralizing strategies seek to stop or reverse the WSLs [10].

Recently, nanohydroxyapatite (n-HAp) has garnered significant interest for its applications in various preventive, curative, and regenerative approaches. Hydroxyapatite (HA) has been considerably applied in tissue engineering, dentin desensitization, bone regeneration, and as a remineralizing substance [11]. The synthetic n-HAp has been proven to have a comparable configuration, construction, and crystallinity as a natural apatite. Additionally, n-HAp may serve as a supply for calcium and phosphate. In acidic conditions, n-HAp can greatly boost the degree of remineralization by facilitating greater ion diffusion in the middle of the demineralized zone. Because of the availability of Ca²⁺ and PO₂⁻⁴ ions, it can restore enamel and inhibit the advancement of early lesions. The nanosized particles, which filled up any tiny pores on demineralized surfaces, act as a framework for calcium and phosphate attraction and deposition from saliva into the enamel’s outermost layer to build a renewed apatite layer [12].

With the advancement in science and research, fluorinated graphene oxide (FG) has been introduced in the market as a novel material derived from graphene oxide. Fluorinated graphene is frequently employed in material design owing to elevated temperature and wear tolerance, corrosion elimination, minimal surface energy, superior mechanical qualities, antimicrobial capability, and anti-demineralization ability because graphene nanosheets were found to be highly beneficial against Streptococcus mutans [13, 14].

Accordingly, this study planned to assess and compare the effectiveness of the fluorinated graphene oxide nanosheets FG and n-HAp with and without microabrasion pretreatment on the cross sectional microhardness (CSMH) and color change of artificially induced WSL. The null hypothesis was that; (1) There was no difference in the subsurface enamel microhardness and Ca/P ratio following remineralization using FG and n-HAp. (2) No color change among the tested remineralizing materials (3) Microabrasion pretreatment had no effect on enamel subsurface microhardness and color change of artificially induced WSPLs.

## Materials and methods

### Sample size estimation

On basis of a previous study results [15] and using G power statistical power analysis (version 3.1.9.4) [16], a total sample size of (*n* = 20; 10 in each subgroup) and (*n* = 10; 5 in each subgroup) was implemented to locate a significant effect size, with a precise power of 0.8 (1-β error) and a degree of significance of 0.05 (5%) (α error) for evaluating the remineralizing effect of nanohydroxyapatite versus fluorinated graphene gel through microhardness and EDX analysis, respectively. While for color change evaluation, based on the previous parameters and a prior study [17], a sample size of (*n* = 10; 5 in each sub group) is estimated.

### Ethical approval and specimens grouping

Before starting the study, the research proposal received acceptance by the Ethical Research Committee, Collage of Oral and Dental Surgery, Misr University for Science and Technology (2024/0026). Thirty bovine mandibles were collected from a regional slaughterhouse (Giza, Egypt). Cryopreservation was performed to preserve the teeth’s physical qualities. Eighty incisors were extracted applying the conventional dental extraction techniques. The soft tissue remnants were mechanically removed away using a scaler. The roots were removed and the enamel surfaces were surveyed under a stereomicroscope to rule out the teeth with cracks, defects or demineralized parts. Facial surfaces were smoothened utilizing silicon carbide papers (180, 600, 1000-grit) then ultrasonically cleaned for 5 min [18]. The teeth were sporadically distributed among 2 groups (*n* = 40) based on the remineralizing agent applied on the artificially induced enamel lesions;

#### Group 1;

Demineralized enamel surface treated with n-HAp paste (1gram of n-HAp (Nano Tech Company for Photo-Electronics, Egypt) dissolved in 1 ml of distilled water [19].

#### Group 2;

Demineralized enamel surface treated with FG oxide gel (prepared in Nano Gate Company, Cairo, Egypt).

Every group was later split into 2 subgroups (*n* = 20), where the specimens of the first subgroup were treated with the remineralizing agent after microabrasion using Opalustre (Ultradent) while in the other subgroup, the specimens were treated with the remineralizing agent without microabrasion.

### Preparation of FG oxide nanosheets

The FG oxide nanosheets were synthesized through hydrothermal method at Nano Gate Company, Cairo, Egypt) [20, 21].

### Characterization and XRD analysis of FG nanosheets

The crystal configuration of manufactured FG was assessed via implementing high-resolution JEOL JEM-2100 Transmission electron microscope (TEM) alongside a 200 kV.G acceleration voltage. The XPERT-PRO Powder Diffractometer equipment was utilized to get an XRD scheme [22].

### FG gel’s preparation

FGO flakes were dispersed in distilled water with sonication and stirring for 1 h; next, 0.6 g of hydroxypropyl methylcellulose (Loba Chemie, India) were gently and progressively sprinkled over the solution at mild temperature along with vigorous stirring to form a homogenous gel.

### Specimens’ preparation and artificial carious lesions

The roots were cut from the teeth utilizing a diamond saw operated at low speed (Hard tissue microtome, Bronwill, E.McGranthinc, MA, USA). Each tooth’s labial enamel surface was coated with an acid-resisting nail polish (Revlon Consumer Products Corporation, New York, USA), with the exception of a 6 × 6 mm part in the center that was wrapped with adhesive tape. The samples were ingrained in acrylic-resin blocks (Acrostone, Anglo Egyptian Company, Egypt) and then split vertically in a buccolingual direction at the center of the window with a diamond saw operated at slow speed (Isomet; Buehler) under continuous water. Two-halves were obtained; one served as an experimental and the other as a self-control specimen to quantify cross-sectional microhardness prior to artificial enamel caries initiation. The self-control specimens’ halves were embedded in resin-blocks having their interior surface oriented upward, then retained in deionized water till they were assessed. Before artificial lesion preparation, each experimental half was encased in a layer of wax (2 mm) with the labial surface exposed. Then the enamel blocks were individually inserted in self-cured polyester resin. Each specimen was immersed for 4 days in a glass container comprised a demineralized solution (2.2 mM CaCl_2_, and 50 mM acetic acid, 2.2 mM KH_2_PO_4_) [23].Each specimen received 2 mm of demineralizing solution for every 1 mm² of exposed enamel [24]. The solution pH was monitored utilizing an electronic pH meter (Deulxe deep vision, model no: 101, California, USA) and corrected with 1 M potassium hydroxide to 4.2 pH. The solution was replaced every day to preclude the solution oversaturation [25].

### Surface treatment of the artificially induced enamel lesions

For the subgroups pretreated using microabrasion prior to the remineralizing agents’ application, a thin Opalustre slurry film was put on the artificial WSL of the experimental halves. Upon moderate to high forces over 60 s, Opalustre slurry was compressed on the tooth surface employing a polishing rubber cup (DENTP Prophylaxis polishing Rubber Cup Latch) in a low-speed handpiece (COXO, Max 30,000 R.P.M.); subsequently, the remineralizing agents were applied. In the other subgroups, the n-HAp paste and FG gel were put on the exposed labial windows in two successive layers by a microbrush and then left for 30 min. The excess materials were removed using a piece of gauze. Thereafter, the remineralizing agents were flushed with deionized water for 5 s. The application of the remineralizing materials was repeated every day. During the 30-day remineralization phase, the specimens were maintained in artificial saliva and incubated at 37 °C. The composition of artificial saliva comprises: KH₂PO₄ (0.3 g/L), methylhydroxybenzoate (2 g/L), sodium CMC (10 g/L), CaCl₂0.2 H₂O (0.166 g/L), K₂HPO₄ (0.8 g/L), and KCl (0.6 g/L) adjusted to pH 6.820. Artificial saliva was substituted with new solution each 24 h to sustain a consistent pH at 7 [26].

### Cross sectional microhardness measurements (CSMH)

CSMH was measured for the self-control halves as well as for the experimental halves following the demineralization and remineralization period. Each specimen was tested by employing 3 equidistant scratches at 50, 100, and 150 μm into the surface over 50 μm distances along a perpendicular line the surface across the split enamel defect. A 100-gram load for 10 s was applied via a microhardness tester (Wilson hardness tester model TUKON 1102 Germany) to accomplish the CSMH estimation [26].

### Elemental analysis using energy‑dispersive x‑ray spectroscopy EDX and scanning Electron microscope SEM

Five specimens from each subgroup were used for elemental analysis; the samples were taken out from the resin blocks and gold-sputter plated before being inspected through a high-vacuum field emission microscope. With an elevated voltage (30 kV) and a magnification varying from 500 to 1000 X, the photographs were captured at 10 mm intervals. The EDX spectrometer combined with the SEM model Prisma E (Thermo Fisher Company, USA) was utilized to quantify the enamel mineral content. The EDX detector provided a histogram plot that illustrated the phosphate and calcium weight ratios. Elemental analysis was carried out before demineralization (at baseline), after demineralization, and following remineralization. In addition to micromorphological scanning at the window location buccally with SEM [26].

### Color change determination

Color assessment were conducted employing a spectrophotometer (VITA Easyshade V, VITA Zahnfabrik, Bad Säckingen, Germany) in compliance with the Commission International de l’Eclariage (CIEDE 2000) values. Color measurements were obtained before demineralization (at baseline), after demineralization, and after remineralization. An indent was drilled on each edge of the resin blocks utilizing a cylindrical bur (MANI, Tochigi, Japan) to standardize the specimens’ position throughout color measurements [24]. Results were calculated by implementing the (L*a*b*c*h*) variables; represented the level of lightness (0 dark, 100 light), the intensity of green/red color, the amount of blue/yellow color, the chroma, and the hue respectively. The overall color change CIEDE2000 (ΔE_00_) was determined for every specimen applying this equation: [27]

$$\begin{gathered}\Delta {E_{00}} = \, \hfill \\\,\,\,\left[ \begin{gathered}\left( {\Delta L\prime \,/\,KLSL} \right)2\, + \,\left( {\Delta C\prime \,/\,KCSC} \right)2\, \hfill \\+ \,\left( {\Delta H\prime \,/\,KHSH} \right)2\, + \,RT\left( {\Delta C\prime \,/\,KCSC} \right)\left( {\Delta H\prime \,/\,KHSH} \right) \hfill \\ \end{gathered} \right]1/2 \hfill \\ \end{gathered} $$

In the context of remineralization, if the ΔE₀₀ value is less than 1.0, there is no visible color change for the human sight. A color change may be visible under controlled settings when the ΔE₀₀ value is between 1.0 and 2.0. Under normal viewing conditions, a color difference between 2.0 and 3.3 is apparent and just noticeable [28].

### Statistical analysis

The statistical analysis was carried out using a software program for Windows (SPSS 20-Statistical Package for Scientific Studies, SPSS, Inc., Chicago, IL, USA).

Numerical data were displayed in means and standard deviations. The Kolmogorov-Smirnov and Shapiro-Wilk tests were implemented to verify the data normality. Means were analyzed using ANOVA, with Bonferroni’s post hoc test for paired comparisons. A paired t-test was served to compare the tested groups. A two-way ANOVA test was utilized to determine the impact of the tested variables and how they interacted. The p-value is two-sided. P-values ≤ 0.05 were deemed significant.

## Results

### Characterization and XRD analysis of FG nanosheets

**Fig. 1:**
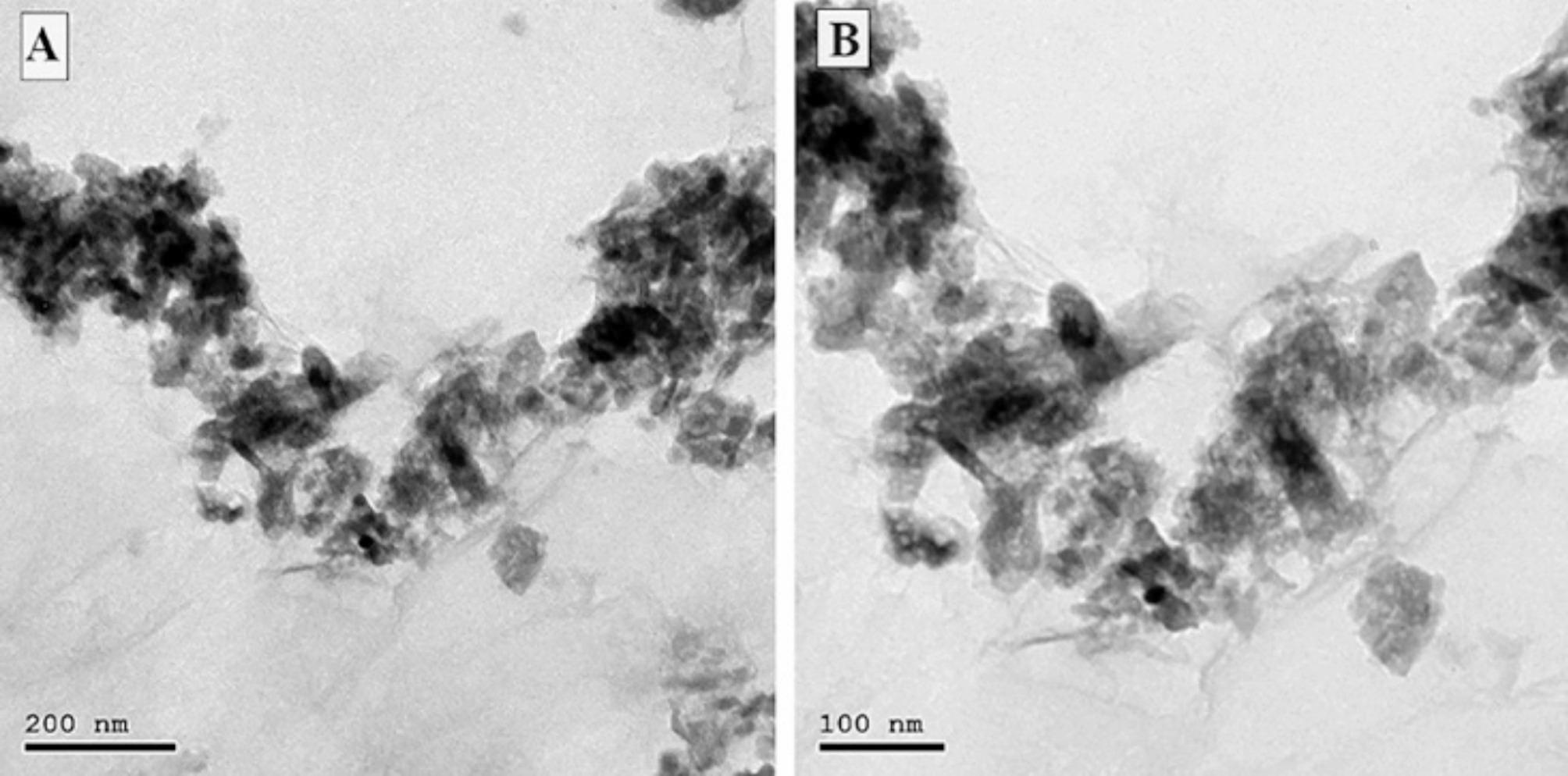
**(A)** Transmission electron microscope (TEM) image of fluorinated graphene oxide nanosheets. **(B)** TEM image of fluorinated graphene oxide nanosheets at higher magnification

**Fig. 2:**
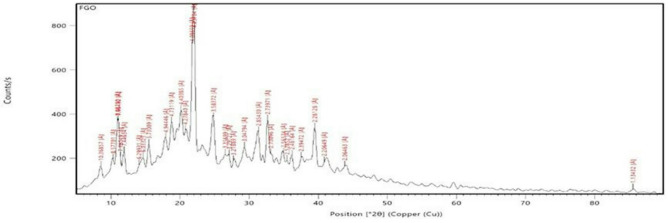
Showing the XRD analysis of the Fluorinated Graphine Oxide nanosheet

TEM exhibited a delicate, translucent two-dimensional FG nanostructure that has transverse measurements ranging from 200 nm to 2 μm. The surface seemed wrinkled and rippled, like graphene Fig. 1. With regard to the XRD scheme, FG dissipated on crystal face (002) but intensified on crystal face (001), suggesting that FG was effectively exfoliated and the existence of a hexagonal crystal layering featuring a substantial fluorine content. Figure 2.

### Color change

Table 1 presented that the highest mean values was recorded in FG (1.88 ± 0.07) followed by n-HAp (1.8 ± 0.06); then (microabrasion + FG) (1.76 ± 0.19) and the lowest value was detected in (microabrasion + n-HAp) (1.71 ± 0.23). The difference between groups was not statistically significant (*p* = 0.114).

**Table 1:** Descriptive statistics and comparison of color ΔE_00_ between groups (ANOVA test)

	Mean	Std. Dev	95% Confidence Interval for Mean		Min	Max	F	*P* value
			Lower Bound	Upper Bound				
Nano-hydroxyapatite	1.80	0.06	1.76	1.85	1.71	1.89	2.13	0.114 ns
Nano fluornated graphene	1.88	0.07	1.83	1.93	1.81	1.99		
Microabrasion + nano hydroxyapatite	1.71	0.23	1.54	1.88	1.30	1.95		
Microabrasion + nano fluornated graphene	1.76	0.19	1.62	1.89	1.42	1.95		

Significance level *p* ≤ 0.05, ns = non-significant

### EDX results

#### Ca / P ratio

Table 2 showed that FG treated specimens exhibited the highest significant mean value (3.29 ± 0.12) followed by n-HAp (2.69 ± 0.18), Microabrasion + FG (2.59 ± 0.07), the control specimens (2.55 ± 0.04), and the Microabrasion + n-HAp treated specimens (2.54 ± 0.04). The demineralized group revealed the least significant value than the other groups (1.78 ± 0.18) (*p* < 0.001). The control, n-HAp, microabrasion + n-HAp, and microabrasion + FG treated specimens were not statistically variant, according to the post hoc test.

**Table 2:** Descriptive statistics and comparison of ca/p ratio between groups (ANOVA test)

Groups	Mean	Std. Dev	95% Confidence Interval for Mean		Min	Max	F	*P* value
			Lower Bound	Upper Bound				
Control	2.55b	0.04	2.52	2.58	2.52	2.62	154.51	0.000*
Demineralized	1.78c	0.18	1.65	1.91	1.51	1.94		
Nano-hydroxyapatite	2.69b	0.18	2.56	2.81	2.54	2.99		
Nano fluorinated graphene	3.29a	0.12	3.20	3.38	3.12	3.43		
Microabrasion with nano hydroxyapatite	2.54b	0.04	2.51	2.56	2.48	2.58		
Microabrasion with nano fluorinated graphene	2.59b	0.07	2.54	2.64	2.52	2.68		

Significance level *p* ≤ 0.05, *significant

Post hoc test: means sharing the same superscript letter are not significantly different

**Fig. 3:**
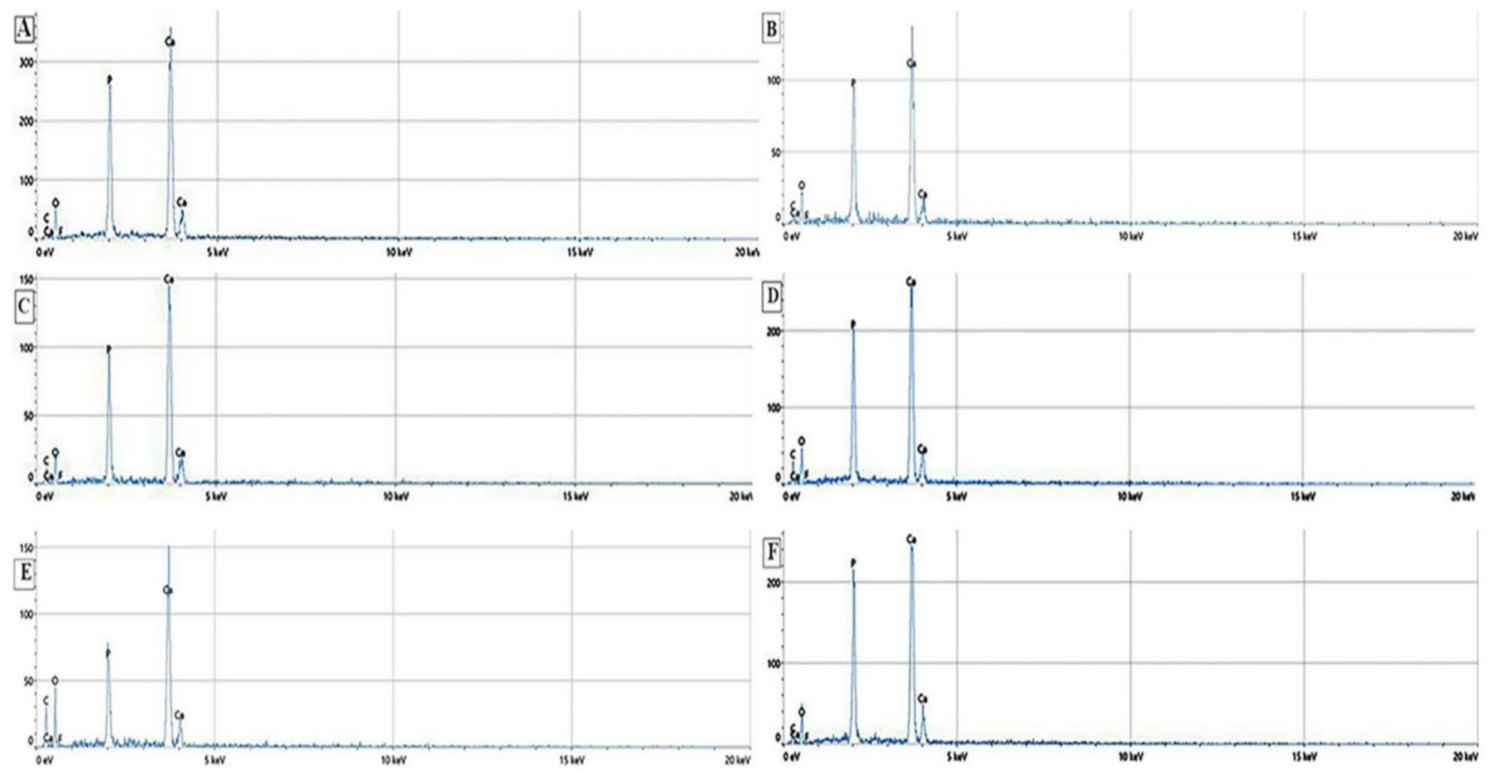
showing the EDX analysis for the untreated enamel (control) **(A)** demineralized enamel **(B)**, Nanohydroxyapatite following microabrasion **(C)**, Nanohydroxyapatite treated enamel **(D)**, FG treated enamel following microabrasion **(E)** and FG treated enamel **(F)**

EDX elemental analysis was demonstrated in Fig. 3 where Fig. 3A depicts the natural enamel composition. The expected peaks are calcium (Ca), phosphorus (P), oxygen (O), and carbon. The peak intensities used as a reference. While demineralized enamel shows a drop in Ca and P peaks due to mineral loss, increased oxygen, and carbon, which reflect the organic matrix Fig. 3B.

In Fig. 3C, microabrasion + n-HAp treated enamel, the Ca and P peaks increased in comparison to Fig. 3B. However, it did not fully reach that of the natural enamel. Figure 3D demonstrate an obvious Ca and P peaks rise in the n-HAp treated enamel compared to the Fig. 3A, indicating more mineral recovery. Figure 3E revealed that Ca and P peaks in enamel remineralized with FG followed by microabrasion were comparable to that of microabrasion + n-HAp remineralized enamel, with increased fluoride element peak. Finally, Fig. 3F there was increased Ca, P, and fluoride peaks implying the superior enamel remineralizing effect of FG.

#### Scanning electron microscope results

**Fig. 4:**
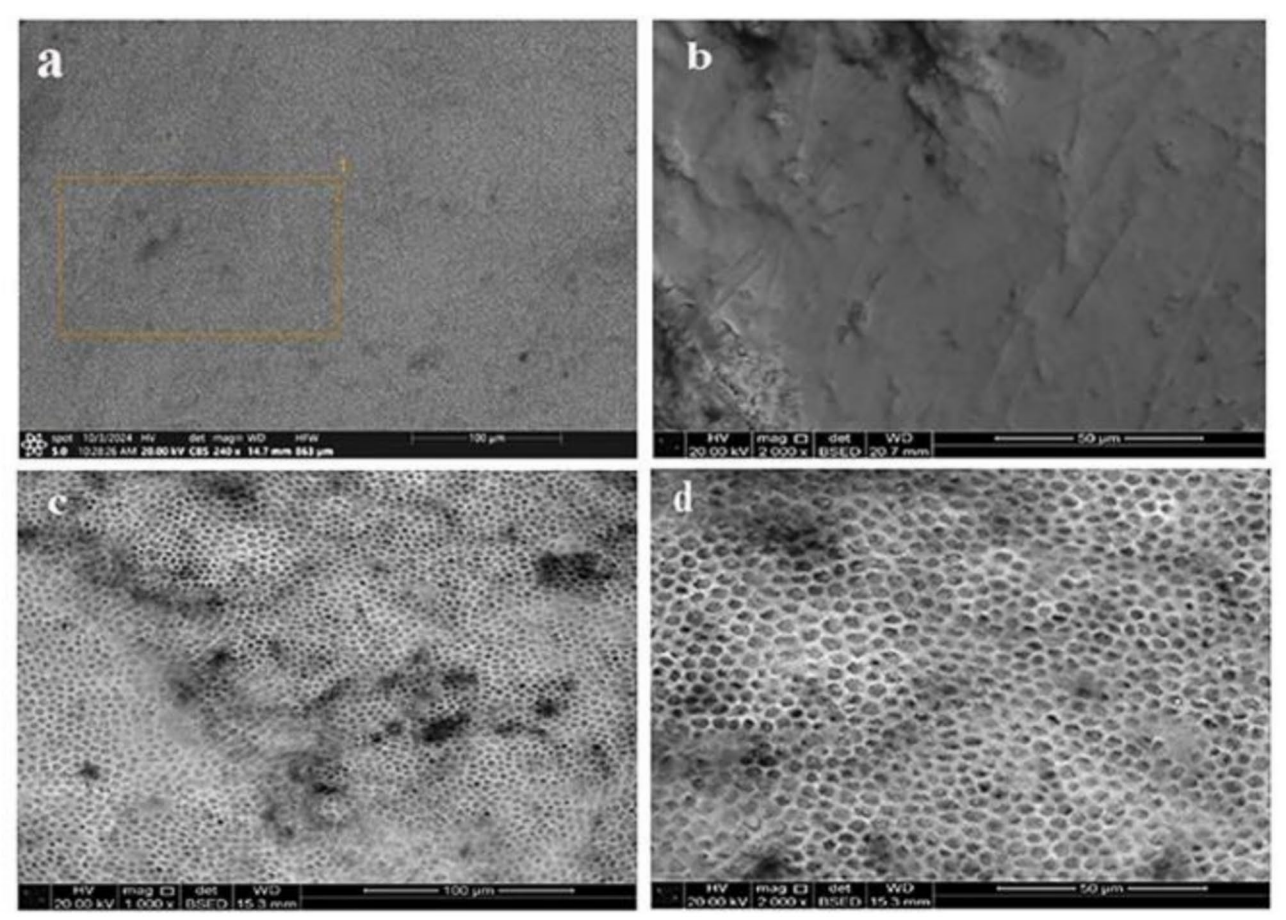
SEM image showing the control and the demineralized enamel surface. (**a** and **b** represent the control at magnification 240 X and 2000 X. respectively. (**c** and **d**) showing the demineralized enamel at magnification 1000 X and 2000 X respectively

SEM images of the control and demineralized enamel surfaces were demonstrated in Fig. 4 Where the SEM micrographs (a & b) represent a healthy enamel structure which appeared relatively smooth with minor irregularities and no significant porosity or evident structural damage. On contrary, demineralized enamel showed increased porosity and surface roughness due to mineral loss. Higher magnification images confirmed the honeycomb-like pattern implying the significant demineralization (c & d).

**Fig. 5:**
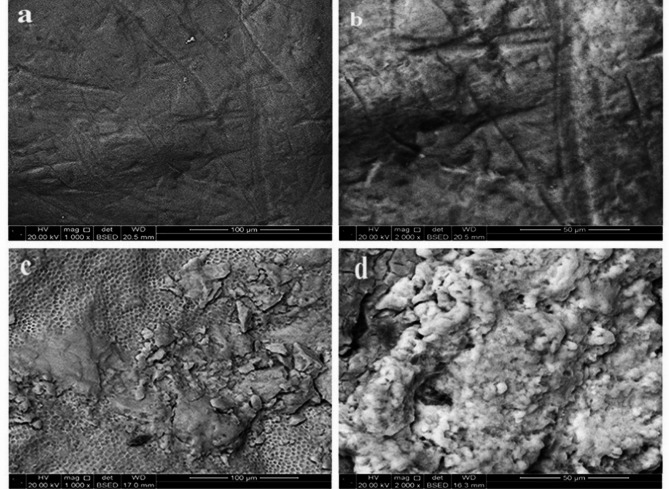
SEM showing the Nanohydroxyapatite treated enamel surface. (**a** and **b**) showing the demineralized enamel surface after treatment with Nanohydroxyapatite after microabrasion at magnification 1000 X and 2000 X respectively. (**c** and **d**) represent the demineralized enamel surface treated with nanohydroxyapatite only at magnification 1000 X and 2000 X respectively

Figure 5a, b. illustrated the demineralized enamel surface following (microabrasion + n-HAp) treatment at magnification 1000 X and 2000 X respectively. The enamel surface appeared smoother compared to demineralized enamel. Fewer pores and irregularities are visible. Some mineral deposits are seen covering the surface, indicating an active remineralization process. At magnification 2000 X, a more compact structure is visible and showing better integration of minerals into the enamel. While Fig. 5c, d represent the demineralized specimens treated with n-HAp at magnifications 1000 X and 2000 X, respectively. The surface is still rough, less uniform and irregular, but high mineral deposits were formed. Compared to Fig. 5b, n-HAp particles were visible as scattered deposits rather than a continuous smooth layer.

**Fig. 6:**
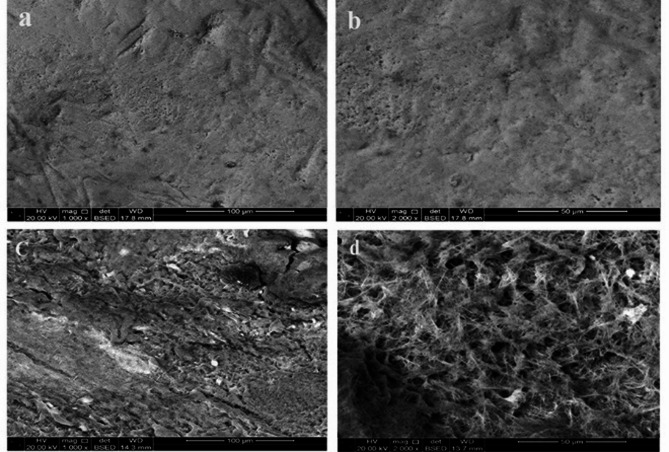
SEM showing the fluorinated graphene oxide nanosheets treated enamel surface. (**a** and **b**) showing the demineralized enamel surface after treatment with fluorinated graphene oxide nanosheets after microabrasion at magnification 1000 X and 2000 X respectively. (**c** and **d**) represent the demineralized enamel surface treated with lfuorinated graphene oxide nanosheets only at magnification 1000 X and 2000 X respectively

The FG treated enamel surface following microabrasion were illustrated in Fig. 6. The SEM micrograph at 1000 X, Fig. 6a, the enamel surface appeared more compact and smooth, suggesting better remineralization with fewer porosities compared to demineralized enamel. At magnification 2000 X (**b**), the structure seemed more integrated. Whereas the SEM micrographs Fig. 6c showed the effect of FG alone on the demineralized enamel at magnification 1000 X. The enamel surface showed high mineral depositions with a compact surface. At magnification 2000 X the enamel surface seemed fibrous, with irregular texture, and suggesting layered mineral depositions influenced by fluoride and graphene oxide interactions.

#### CSMH results

Table 3 illustrated that in the control group showed no significant differences at 50,100, and at 150 μm [(*p* = 0.195), (*p* = 0.995), and (*p* = 0.545)] respectively. Similarly, the demineralized group, there was no significant differences at 50, 100, and 150 μm [(*p* = 0.088), (*p* = 0.101), and (*p* = 0.995)] respectively.

**Table 3:** Descriptive statistics and comparison of microhardness between groups and between different depths (thickness) in the same group (ANOVA test)

Groups	Groups	50 μm		100 μm		150 μm		*P* value (between different depths
		Mean	Std. Dev	Mean	Std. Dev	Mean	Std. Dev	
Control	Nano-hydroxyapatite	105.40 C	5.15	172.80B	4.89	226.40 A	10.84	0.000*
	Nano fluorinated graphene	103.80 C	2.97	172.30 B	4.83	226.10 A	4.28	0.000*
	Microabrasion + nano hydroxyapatite	106.00 C	5.29	172.30 B	4.83	230.50 A	6.43	0.000*
	Microabrasion + nano fluorinated graphene	108.90 C	6.82	172.30 B	5.48	227.40 A	6.82	0.000*
	*P* value (between groups)	0.195 ns		0.995 ns		0.545 ns		
Demineralized	Nano-hydroxyapatite	67.24 C	4.63	103.80 B	2.97	172.80 A	4.89	0.000*
	Nano fluorinated graphene	70.80 C	3.68	106.00 B	5.29	172.30 A	4.83	0.000*
	Microabrasion + nano hydroxyapatite	70.30 C	1.64	103.60 B	5.06	172.30 A	4.83	0.000*
	Microabrasion + nano fluorinated graphene	69.40 C	2.12	108.90 B	6.82	172.30 A	5.48	0.000*
	P value (between groups)	0.088 ns		0.101 ns		0.995 ns		
Remineralized	Nano-hydroxyapatite	92.30d C	8.86	168.80 b B	8.39	227.40 c A	6.82	0.000*
	Nano fluorinated graphene	98.44c C	3.22	191.70 a B	5.85	234.40 a A	3.75	0.000*
	Microabrasion + nano hydroxyapatite	112.54a C	12.76	167.50 d B	7.93	229.20 b A	4.05	0.000*
	Microabrasion + nano fluorinated graphene	106.93b C	9.53	168.30c B	5.64	226.70 d A	5.56	0.000*
	P value (between groups)	0.000*		0.000*		0.000*		

Significance level *p* ≤ 0.05, *significant, ns = non-significant

Post hoc test: Within the same column, values with different small superscript letters are significantly different. Within the same row, values with different capital superscript letters are significantly different

In the remineralized group at 50 μm, Microabrasion + n-HAp had the highest significant value (112.54 ± 12.76) (*p* < 0.001). At 100 and 150 μm, FG showed the highest significant value (191.7 ± 5.85), (234.4 ± 3.75) respectively. Alternatively, in all groups, the microhardness values recorded at 150 μm were the greatest, while those at 50 μm were the lowest, with a remarkable statistical difference between the examined depths (*p* < 0.001).

## Discussion

In view of the current study results, the null hypothesis was rejected as there was a difference in the subsurface enamel microhardness and color change among the tested remineralizing materials in conjunction with or without microabrasion pretreatment.

WSLs are indicative characteristics of demineralization beneath the apparent healthy enamel, resulting in a decline of the translucency, hardness, and typical surface qualities. Several approaches have been proposed to stop and obscure these lesions [15]. Furthermore, additional investigations are required to examine the effectiveness of various approaches in modifying the enamel mineral composition. So, the current study planned to assess and compare the effectiveness of the fluorinated graphene oxide nanosheets FG and n-HAp with and without microabrasion pretreatment on the cross sectional microhardness (CSMH) and color change of artificially induced WSL.

The study assessed enamel microhardness at various depths (50, 100, and 150 μm) based on previous investigations which found that the deepest layer of the lesion was 150 μm using the same demineralizing solution and period, resulting in lesions with defined depths for study [29, 30]. Quantifying the color change induced by demineralization followed by remineralization is critical for determining enamel’s esthetic success and functional recovery. Making it an important criterion in dental research and clinical practice [31–33].

The current study used the CIEDE2000 (CIE 2000) formula rather than the CIELAB (CIE 76) model, as this provides a more accurate and perceptually consistent evaluation of color variations, particularly in low-chroma areas such as enamel. By compensating for brightness, chroma, and hue interactions, CIE 2000 is more in line with human visual perception, making it excellent for identifying small color changes in dental research [28].

The color change between demineralized and remineralized enamel is due to structural changes affecting light reflection. Demineralization creates micro porosities that scatter light, causing a white, opaque appearance. Remineralizing agents help refill these pores, restoring translucency and improving color [34, 35].

In the current study, FG demonstrated the highest mean color improvement, followed by n-HAp and microabrasion with the both tested nanomaterials. The CIEDE2000 values across all groups ranged from 1.30 to 1.99, indicating that all treatments produced color changes that were near or slightly above the perceptibility threshold (ΔE_00_ ≈ 1.0) and within or near the clinical acceptability threshold (ΔE_00_ ≈ 1.8).

These results suggest that FG may have a slightly superior effect on esthetic improvement due to its strong affinity for enamel and potential to enhance surface mineralization. Conversely, the combination treatments, particularly microabrasion + n-HAp, showed more variable results and lower mean values. The differences did not reach statistical significance; which may favor using these nanomaterials alone over their combination with microabrasion.

FG revealed a higher performance in improving the enamel color owing to its unique structural and optical features. FG is a graphene derivative that possesses fluorine atoms that are covalently attached to the carbon framework, changing the carbon bonds from sp² to sp³ orientation [36].Graphene has distinct optical features, including full-color emission, which could improve the aesthetic look of the enamel by increasing its translucency and brightness. These changes result in enhanced light-emitting abilities, which contributed to better aesthetic outcomes when applied to enamel surfaces [37]. Additionally, the two-dimensional structure of graphene allows greater integration into dental materials, resulting in increased surface smoothness and gloss. Finally, color enhancement for long term could be an advantage due to the antibacterial activity of FG as it could reduce the colonization of bacteria on the enamel surface [38].

Regarding n-HAp, it is a biocompatible material which closely matches the bone and teeth’s mineral component. It could successfully remineralize the demineralized enamel and improve its surface characteristics. Also previous study found that n-HAp can minimize enamel surface roughness and improve color stability following orthodontic procedures [39].

Based on the present study results, microabrasion before remineralizing material application didn’t show color enhancement compared to the remineralizing material alone. It could be explained by the fact that microabrasion removes a thin layer of the tooth’s outer demineralized or stained surface. However, it improves the enamel’s smoothness it does not address the optical qualities of the underlying enamel structure as efficiently as remineralization does. As a thin layer of enamel is removed, it may expose a less-optically favorable layer beneath, potentially leading to reduction in the tooth surface translucency and refractive index [40].

Furthermore, biomimetic remineralizing agents help to restore the natural translucency of the enamel as remineralization may not fully replace damaged enamel following microabrasion, resulting in a tooth that appears more opaque or less translucent than remineralization alone on untreated enamel [41]. This argument is consonant with a previous study, investigated the effect of microabrasion combined with remineralization on enamel color and mineral content, implying that microabrasion may impede full recovery of enamel color and structure following enamel loss [40].

However, this explanation contradicts the findings of an earlier study, which concluded that microabrasion followed by remineralization, increased both enamel microhardness and color stability [41]. The diverse results in the current study might be correlated to the demineralized enamel, whereas the earlier study used sound enamel. The baseline enamel state has an impact on the efficiency of microabrasion and remineralization. Demineralized enamel may not respond as effectively to remineralization and color restoration as sound enamel.

Regarding EDX analysis, FG alone demonstrated improved remineralization compared to the other treatments. This could be explained by the fact that FG contains fluoride, which created fluorapatite that has a higher Ca/P ratio than hydroxyapatite and is less susceptible to acid breakdown [42]. Furthermore, the nano size of FG permitted efficient integration into the enamel structure, resulting in a more stable mineralized surface [43]. Moreover, graphene-based materials could improve the ionic exchange with calcium and phosphate deposition, resulting in better remineralization efficiency compared to n-HAp which imitated the natural enamel composition, but without fluoride, its Ca/P ratio maintains within normal enamel values [42, 44].

Also, Microabrasion removes the outer enamel layer, which is considering the lowest Ca/P ratio. In demineralized lesion leaving the hard surface and compress the remaining demineralized enamel on this surface this could be the reason why Ca/P ratio close to the control ratio [44].

The CSMH assessment was selected as it assess the microhardness both superficially and at different enamel depths which could outline the difference between the surface repair and the true remineralization.

At 50 μm depth, that microabrasion along with the remineralizing material had a higher microhardness as microabrasion removed the superficial demineralized enamel, compressing and compacted the surface, which suggested a surface treatment not a true remineralization of the lesion [45, 46].

At depth 100 and 150 μm; FG showed the highest CSMH as fluoride integration enhances fluorapatite formation, which is improves the hardness. Graphene’s high surface area allows deeper calcium and phosphate deposition into enamel [43].

However, n-HAp showed improvement similar to the sound enamel. Since hydroxyapatite primarily filled the superficial defects, the remineralization at this depth was effective and managed to restore microhardness closer to normal enamel however, it may not penetrate as deeply as fluorinated materials [44].

Additionally, FG was utilized in a gel form which allowed better flow, adherence, uniform distribution, penetration and interaction with the enamel’s microstructure. In contrast, varnishes tend to create a more superficial coating on the enamel, with limited penetration into deeper micro-irregularities [47, 48].

From the limitations encountered in this study were the assessment of the color stability, the acid- dissolution resistance of the remineralized enamel lesions, and the assessment of other elements in EDX analysis as Na, Mg ions for better evaluation of the remineralizing potentials of the proposed treatments. In addition, further clinical investigations are essential to assess the effect of the tested materials.

## Conclusions

### Under the limitation of the study it was concluded that

1. FG provides greater aesthetic enhancement and remineralization, most likely due to fluoride incorporation and increased enamel integration.
2. n-HAp is a promising biomimetic material for enamel remineralization, especially as it resembles the natural enamel structure.
3. Microabrasion provides a superficial increase in enamel smoothness and hardness, while as a pretreatment before remineralization has little effect on improving cross sectional microhardness.

## Data Availability

The data sets used and/or analyzed in the current research are available upon reasonable request from the corresponding author.

## Abbreviations

FG: Fluorinated Graphene Oxide Nanosheets
n-HAp: Nano-Hydroxyapatite
CSMH: Cross-Sectional Microhardness
WSL: White Spot Lesions
EDX: Energy-dispersive X-ray spectroscopy
SEM: Scanning electron microscopy
